# Low-Temperature Infrared Spectra and Ultraviolet-Induced Rotamerization of 5-Chlorosalicylaldehyde

**DOI:** 10.1021/acs.jpca.2c03685

**Published:** 2022-07-29

**Authors:** Anna Luiza B. Brito, José P. L. Roque, İsa Sıdır, Rui Fausto

**Affiliations:** † CQC-IMS, Department of Chemistry, University of Coimbra, 3004-535 Coimbra, Portugal; ‡ Department of Physics, Bitlis Eren University, 13000 Bitlis, Turkey

## Abstract

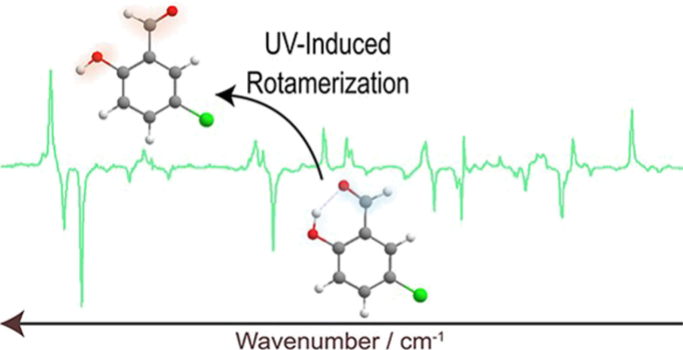

5-Chlorosalicylaldehyde (abbreviated as 5CSA) is an important chemical used in the synthesis of fragrances, dyes, and pharmaceuticals. In this investigation, 5CSA isolated in solid N_2_, at 10 K, and in its neat amorphous and crystalline phases, at 50 and 190 K, respectively, were investigated by infrared spectroscopy and DFT(B3LYP)/6-311++G(d,p) calculations. The systematic theoretical analysis of the 5CSA conformational landscape showed that the compound exhibits four different conformers, which were structurally characterized in detail. In the as-deposited low-temperature matrices of 5CSA, only the most stable conformer, the intramolecularly hydrogen-bonded form **I**, was found. The same was observed in the case of the investigated low-temperature amorphous and crystalline phases of 5CSA. Conformer **I** was successfully converted into a higher-energy conformer(**II**), where both aldehyde and hydroxyl groups are rotated by 180° relative to their position in the initial conformer, through narrowband ultraviolet (UV) (λ = 308 nm) in situ irradiation of the as-deposited N_2_ matrix of 5CSA. The infrared spectra of both matrix-isolated conformers, as well as those of the neat amorphous and crystalline phases of 5CSA, were assigned and interpreted in comparative terms, allowing us to elucidate structurally and vibrationally relevant effects of the main intra- and intermolecular interactions operating in the different studied phases. Very interestingly, the observed UV-induced **I** → **II** rotamerization was found to take place in an exclusive basis, with no other photochemical processes being observed to occur upon UV irradiation, under the experimental conditions used in the present investigation.

## 1 Introduction

Salicylaldehyde (2-hydroxybenzaldehyde) is a well-known compound, which receives major applications in the perfume industry.^1,2^ The compound occurs in nature in buckwheat,^3^ and it is also one of the components of castoreum, the exudate from castor sacs of mature beavers.^4^ Castoreum has been used in perfumery for a long time, as well as in the food industry.^5^ Salicylaldehyde and its derivatives are key precursors of many important chelating agents, and additionally, they are used in the synthesis of Schiff bases for different applications.^6−8^

Salicylaldehyde is the smallest aromatic molecule displaying excited-state intramolecular proton transfer (ESIPT), which takes place in less than 100 fs, yielding (6*Z*)-6-(hydroxymethylidene)cyclohexa-2,4-dien-1-one.^9^ Mostly because of this, the compound has been extensively studied regarding its photochemistry, and it was found that, besides ESIPT, the molecule undergoes other competing ultraviolet (UV)-induced transformations, specifically rotamerization about the exocyclic C–O(H) and C–C(HO) bonds, decarbonylation, and generation of ketene species.^9−12^

In this study, the 5-chloro-derivative of salicylaldehyde, 5-chlorosalicylaldehyde (abbreviated as 5CSA), has been investigated. This compound is an important chemical used in the synthesis of fragrances and dyes^13^ and also in the production of pharmaceuticals, such as imine resveratrol derivatives applied as multitargeted agents against Alzheimer’s disease.^14,15^ In spite of its practical relevance, it has only been scarcely studied previously.

The crystalline structure of 5CSA is known,^16,17^ the crystals being monoclinic, space group *P*2_1_ (*n*° = 4), with *a* = 3.8818(5) Å, *b* = 5.6515(7) Å, *c* = 15.204(2) Å, β = 93.176(1)°, at room temperature, and two molecules per unit cell (*Z* = 2).^17^ In the crystals, the molecules adopt a conformation where the hydroxyl group is intramolecularly hydrogen bonded to the aldehyde oxygen atom and are arranged in two alternate arrays that make an angle of ca. 60° between them; they are further interconnected by intermolecular O–H^···^O hydrogen bonds, forming zig-zag chains.^16,17^ To the best of our knowledge, no other structural, vibrational, or photochemical studies (either experimental or theoretical) have been reported on 5CSA hitherto.

In the present investigation, 5CSA has been isolated in solid N_2_, at 10 K, and characterized structurally and vibrationally. The intramolecularly hydrogen-bonded conformer of the molecule was found to be the only species present in the as-deposited matrices. This conformer was successfully converted into a higher-energy conformer, where both aldehyde and hydroxyl groups are rotated by 180° relative to their position in the initial conformer, through narrowband UV (λ = 308 nm) in situ irradiation. The infrared (IR) spectra of both conformers were assigned with help of DFT(B3LYP) vibrational calculations, which were further used to perform a full exploitation of the conformational landscape of the molecule, allowing for the identification of two additional conformers of higher energy. Very interestingly, besides rotamerization, no other photochemical processes were observed to take place upon UV irradiation under the experimental conditions used in the present investigation. Finally, the low-temperature IR spectra of the amorphous compound and of its crystalline state, at 50 and 190 K, respectively, were also obtained and interpreted.

## 2 Computational Details

Calculations were undertaken using Gaussian 09 (revision A.02)^18^ at the DFT(B3LYP)/6-311++G(d,p)^19−24^ level of theory. The optimized structures of all stationary points described in this study were confirmed to correspond to true minimum or first-order transition states by analysis of the corresponding Hessian matrix.

Harmonic IR spectra were obtained at the same level of theory and used to simulate the spectra shown in the figures, through convolution with Lorentzian functions having a full-width-at-half-maximum equal to 1.5 cm^–1^. The calculated wavenumbers were scaled by 0.983 and 0.955, below and above 1800 cm^–1^, respectively, in order to account for the incomplete nature of the basis set, approximated theoretical method and, mostly, anharmonicity. The vibrational analysis was performed with help of the vibrations’ animation module of Chemcraft (version 1.8).^25^ Potential energy distribution (PED) was obtained by transforming the computed force constants with respect to Cartesian coordinates into the force constants with respect to internal coordinates, which allowed the PED analysis to be carried out, as described elsewhere.^26^ The internal coordinates used were defined as recommended by Pulay et al.^27^ and are given in Table S1. The atom numbering used for the definition of the internal coordinates is shown in Table S2.

Tunneling rates for the isomerizations were estimated using the Wentzel–Kramers–Brillouin (WKB) model for a parabolic barrier. The reaction barrier shapes (heights and widths), required for the WKB predictions, were obtained from the energy profiles along the Cartesian (not mass-weighted) intrinsic reaction coordinates (IRCs), as described elsewhere.^28^

## 3 Experimental Methods

5-Chlorosalicylaldehyde was obtained commercially (purity >98%) and used without further purification. The matrices were prepared by codeposition of the sublimed compound and N_2_ (N60; Air Liquide) onto a CsI substrate attached to the cold (10 K) tip of an APD Cryogenics DE-202A closed-cycle helium refrigerated cryostat. The proper isolation of the molecules of the compound was confirmed by inspection of the IR spectra, which do not show evidence of the presence of aggregates. The compound was sublimed (∼308 K) using a specially designed home-made variable temperature Knudsen cell, which was attached to the cryostat through a needle valve, and the valve nozzle was kept at room temperature (298 K). The temperature of the cold CsI window was measured directly at the sample holder by a silicon diode sensor using a digital temperature controller (LakeShore 335), with an accuracy of 0.1 K.

IR spectra were recorded using a Thermo Nicolet 6700 FTIR spectrometer (0.5 cm^–1^ spectral resolution), equipped with a mercury cadmium telluride (MCT-B) detector and either a Ge/KBr beam splitter for mid-IR measurements (4000–400 cm^–1^; 0.5 cm^–1^ spectral resolution) or a CaF_2_ beam splitter for near-IR measurements (7500–4000 cm^–1^; 1.0 cm^–1^ spectral resolution). The instrument was continuously purged by a stream of dry, CO_2_-filtered air, in order to avoid interference of atmospheric H_2_O and CO_2_.

UV and near-IR irradiations of the matrices were carried out using tunable narrowband (∼0.2 cm^–1^ spectral width) light provided by the frequency-doubled signal (UV range) or idler (near-IR range) beam (pulse energy ∼1 mJ) of a Spectra Physics Quanta-Ray MOPO-SL optical parametric oscillator (OPO) pumped with a pulsed Nd:YAG laser (repetition rate = 10 Hz, duration = 10 ns). The outcome of the performed irradiations was monitored by collecting IR spectra.

## 4 Results and Discussion

### 4.1 DFT Calculations: Conformers, Relative Energies, and Barriers for Conformational Isomerization

**Figure 1:**
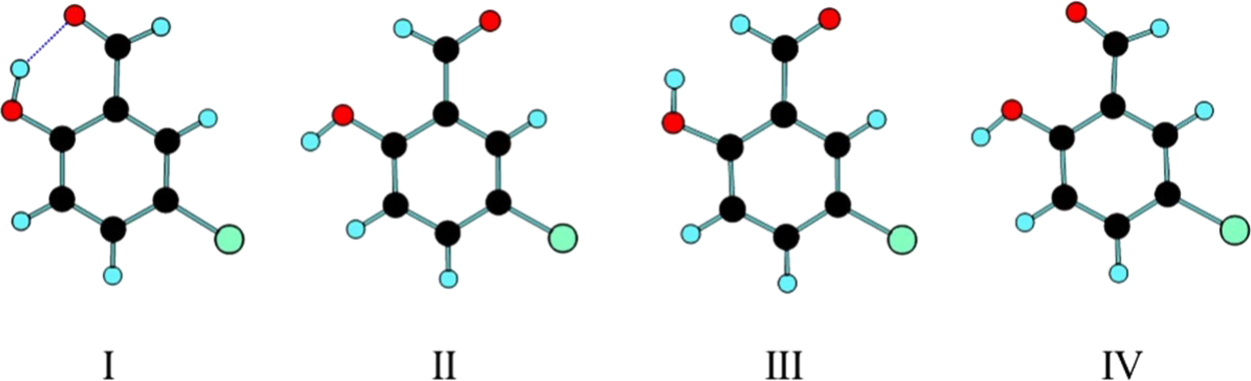
Conformers of 5CSA, as predicted by the DFT(B3LYP)/6-311++G(d,p) calculations.

The molecule of 5CSA has two conformationally relevant internal degrees of freedom, which correspond to the internal rotations around the exocyclic C–C and C–O bonds connecting the aldehyde and hydroxyl groups to the aromatic ring. Four different conformers were identified on the potential energy surface of the molecule at the B3LYP/6-311++G(d,p). These conformers are represented in Figure 1, while their calculated relative electronic energies (without and with inclusion of zero-point vibrational energy), standard Gibbs energies (298 K), and estimated room-temperature gas-phase equilibrium populations are given in Table 1. The calculated geometries of the different conformers (Cartesian coordinates) are provided in the Supporting Information (Table S2).

**Table 1:** Relative Electronic Energies without (Δ*E*_el_) and with (Δ*E*_ZPE_ = Δ*E*_el_ + ΔZPE) Zero-Point Vibrational Energy Correction (ΔZPE), Standard Gibbs Free Energy (Δ*G*°), and Estimated Gas-Phase Room-Temperature Equilibrium Populations (*Pop.*) of the Conformers of 5CSA^a^

	I	II	III	IV
Δ*E*_el_	0.0	33.9	39.8	45.9
Δ*E*_ZPE_	0.0	31.1	36.9	43.2
Δ*G*°	0.0	28.9	34.6	41.2
*Pop.* (%)^b^	99.999	0.001	8 × 10^–5^	6 × 10^–6^

a Energies in kJ mol^–1^ (see Figure 1 for structures of the conformers).

b The populations are given assuming that the Gibbs energies are not affected by any error, thus with an exaggerated accuracy; from the physical point of view, what these numbers allow us to conclude is that conformer **I** is essentially the sole form present in the room-temperature gas-phase equilibrium.

As expected, the most stable conformer of 5CSA (conformer **I**) bears a strong stabilizing O–H^···^O= intramolecular hydrogen bond (*d*_O···O_ = 2.640 Å; *d*_H···O_ = 1.771 Å; ∠O–H^···^O = 145.4°, values computed for the isolated molecules). Because of this interaction, conformer **I** is more than 30 kJ mol^–1^ lower in energy than all the other conformers (see Table 1), which translates in practical terms to being the only form existing in the gas phase at room temperature.

Conformer **II** has a relative energy of 31.1 kJ mol^–1^ (zero-point corrected energy) and has both the aldehyde and hydroxyl groups rotated by 180° relative to their position in the most stable conformer. In this conformer, two bond-dipole/bond-dipole stabilizing interactions are present, which are established between the nearly antiparallelly aligned bond-dipoles of the C–O(H) and C–H (aldehyde) bonds, on the one side, and of the C=O and *ortho* C–H bonds, on the other side (see Figure 1). These two stabilizing intramolecular interactions are responsible for the lower energy of this conformer compared to those of conformers **III** and **IV** (36.9 and 43.2 kJ mol^–1^, respectively). In conformer **III**, the stabilizing bond-dipole/bond-dipole interaction involving the C=O and *ortho* C–H bonds is still present, but the C–O(H)/C–H (aldehyde) bond-dipole/bond-dipole interaction that is present in conformer **II** is replaced by a repulsive interaction between the hydroxyl and aldehyde hydrogen atoms, which justifies the higher energy of conformer **III** compared to conformer **II**. Conformer **IV**, the highest energy form of 5CSA, has its relative energy determined by the repulsive interactions between the two oxygen atoms (hydroxyl and aldehyde) and between the aldehyde hydrogen and the ring hydrogen atom in the *ortho* position to the aldehyde group (see Figure 1).

Due to the distinct intramolecular interactions, the different conformers of 5CSA have different geometries of the structurally most relevant moieties, i.e., the aldehyde and hydroxyl groups, while the C–Cl bond length is predicted by the calculations to be nearly identical in all the forms (1.758 Å in conformers **I** and **II** and 1.757 Å in **III** and **IV**). The calculated C=O bond length is considerably larger in conformer **I** (1.227 Å), where the aldehyde oxygen atom acts as the H-bond acceptor in the intramolecular hydrogen bond, than in the remaining forms (1.207–1.213 Å), while the C–H aldehyde bond length is shorter in conformers **I** and **II** (1.105 and 1.103 Å, respectively) than in conformers **III** and **IV** (1.114 and 1.112 Å), due to the H^···^H repulsions existing in these latter forms. Moreover, the C–C bond connecting the aldehyde group to the aromatic ring is considerably shorter in conformer **I** (1.456 Å) than in the other conformers (1.474, 1.483, and 1.484 Å, in conformers **II**, **III**, and **IV**, respectively), demonstrating that this bond has a higher double bond character in the most stable conformer; that is, conformer **I** bears a more extensive π-electron delocalization involving the aldehyde moiety and the aromatic ring. Conformer **II** has an intermediate C–C bond length, i.e., an intermediate level of π-electron delocalization, while conformers **III** and **IV** have identical and longer C–C bond lengths in result of the smallest π-electron delocalization they exhibit among all 5CSA conformers. Regarding the geometry of the hydroxyl group, the calculations indicate that the O–H bond length in conformer **I** (0.984 Å) is much longer than those in the remaining conformers (0.963–0.964 Å), as expected due to the participation of the OH group of conformer **I** in the O–H^···^O intramolecular hydrogen bond. In addition, the intramolecular H-bond also determines a considerably shorter C–O bond in conformer **I** (1.339 Å) as compared to those calculated for the non-hydrogen-bonded conformers (1.352–1.364 Å). Indeed, if one thinks in terms of Lewis structures and take into account the intramolecular H-bond, it can be envisaged that an enol-type structure contributes to the overall structure of conformer **I**, which can justify the shorter C–O and longer C=O bonds in conformer **I** compared to the remaining conformers.

**Figure 2:**
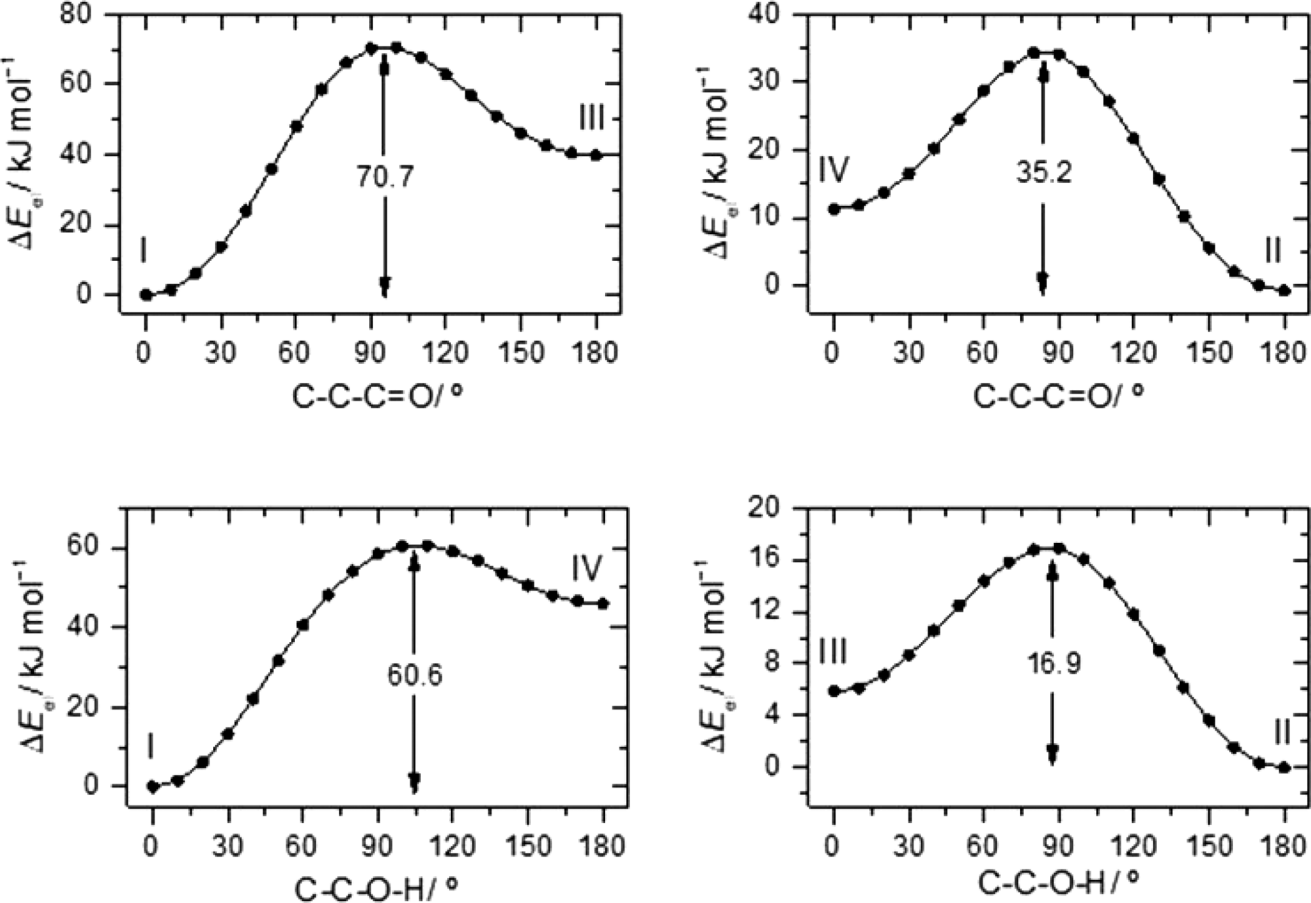
DFT(B3LYP)/6-311++G(d,p) calculated potential energy profiles for conformational isomerization in 5CSA (internal rotation around the C–C(HO) and C–O bonds). The dihedrals (scan coordinates) were defined including the ring carbon atoms to where the hydroxylic and aldehyde substituents are connected. The values of the barriers counted from the higher energy conformer are **III** → **I**, 30.9 kJ mol^–1^; **IV** → **II**, 23.2 kJ mol^–1^; **IV** → **I**, 14.7 kJ mol^–1^; and **III** → **II**, 11.0 kJ mol^–1^. For each layer, the relative zero energy was assumed to be the most stable conformer involved in the isomerization reaction.

The potential energy profiles for ground-state interconversion between the 5CSA conformers was calculated by performing relaxed potential energy scans along the relevant isomerization coordinates (Figure 2).

The data presented in Figure 2 demonstrate that all conformers are located in well-defined minima, with appreciably high energy barriers separating them from other forms. The highest energy conformer **IV** can be directly transformed into forms **I** (through rotation of the OH group) and **II** (via rotation of the aldehyde moiety), and the corresponding barriers amount to 14.7 and 23.2 kJ mol^–1^, respectively. Conformer **III** conversion into **I** (aldehyde rotation) and **II** (OH rotation) can take place through barriers, which amount to 30.9 and 11.0 kJ mol^–1^, respectively. Since conversion of conformer **II** into conformer **I** requires the rotation of both aldehyde and hydroxyl groups, it can occur step wisely either via form **III** or via conformer **IV**, in the first case with a global barrier of 30.9 kJ mol^–1^ (second step; **III** → **I**) and in the second case with an overall barrier of 35.2 kJ mol^–1^ (first step; **II** → **IV**). It is, however, very much probable that in both cases, the **II** → **I** isomerization occurs with the second step starting before the first one being completed, so that the effective ground-state barrier for this isomerization shall be somewhat smaller than 30 kJ mol^–1^. In any case, the **II** → **I** rotamerization always involves a substantial movement of the aldehyde oxygen atom, and the isomerization barrier is certainly high enough to prevent spontaneous conversion of **II** into **I** via quantum mechanical tunneling at low temperatures, so that conformer **II** can be expected to be stable under low-temperature matrix isolation conditions if it can be generated in situ by any method (under these conditions, the over-the-barrier process is certainly not accessible either). As shown in Section 4.3, this expectation could be confirmed in the present investigation, with conformer **II** being produced by in situ UV excitation of conformer **I** previously trapped into a N_2_ matrix (10 K) from the gas phase of the compound. On the other hand, the barriers for internal rotation of the OH group in conformers **III** and **IV** (11.0 and 14.7 kJ mol^–1^, respectively; see Figure 2), which involves only movement of the light hydroxyl hydrogen atom, are low enough to allow fast decay of these two conformers into conformers **II** and **I**, respectively, by H-atom tunneling. Hence, conformers **III** and **IV** should be considered fleeting species, whose experimental detection by steady-state spectroscopic methods does not seem to be achievable, even under low-temperature matrix isolation conditions.^29^

### 4.2 IR Spectra of Matrix-Isolated 5CSA

**Figure 3:**
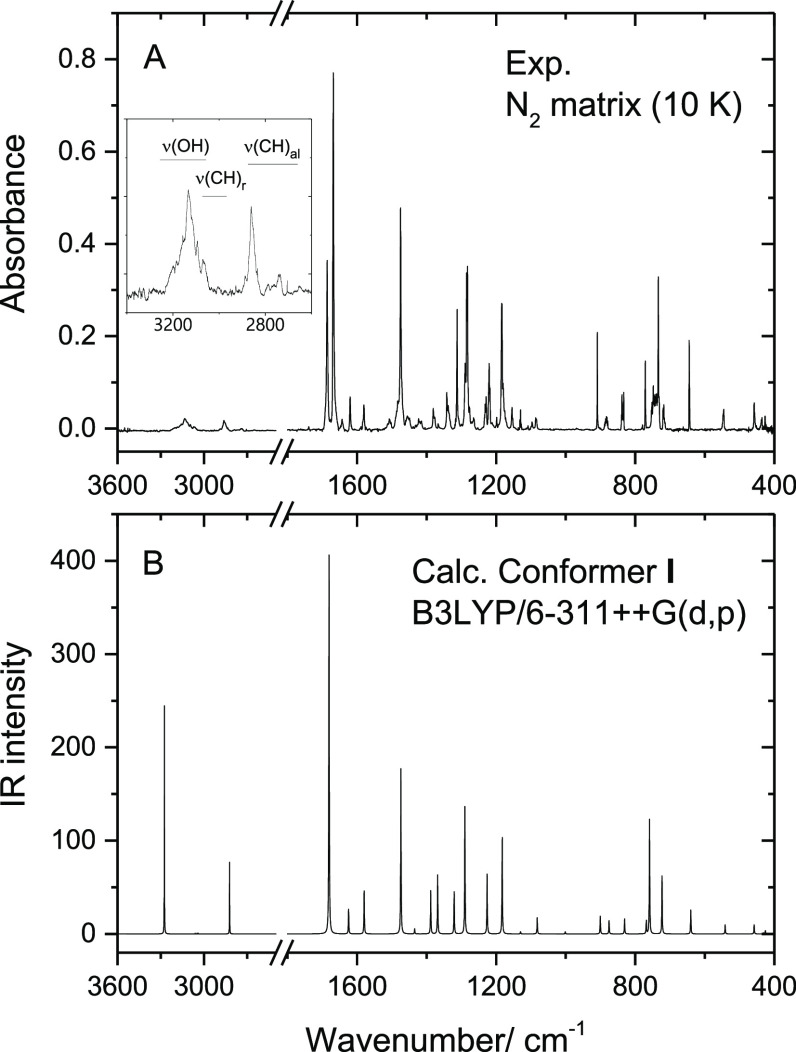
(A) Experimental IR spectrum of 5CSA isolated in a N_2_ matrix, at 10 K, and (B) simulated IR spectrum of conformer **I** of the molecule, built using the B3LYP/6-311++G(d,p) calculated vibrational data (see Section 3 and Table 2 for details). ν(CH)_r_ and ν(CH)_al_ refer to CH stretching modes of the C–H bonds of the ring and of the aldehyde group, respectively.

Molecules of 5CSA were isolated in a N_2_ matrix, at 10 K, as described in Section 3. The IR spectrum of the prepared matrix is presented in Figure 3 and compared with the simulated IR spectrum of conformer **I**, which was built from the B3LYP/6-311++G(d,p) calculated vibrational data (see Section 3 for details).

The predicted spectrum for conformer **I** matches very well the experimental spectrum of 5CSA displayed in Figure 3. It is clear that only conformer **I** is present in the as-deposited matrix, as it could be anticipated taking into account the relative energies of the 5CSA conformers. Though this conclusion can be extracted from the detailed analysis of the whole spectrum, the high-frequency region is particularly clear regarding this point. In fact, among the conformers of 5CSA, only the most stable intramolecularly hydrogen-bonded conformer **I** is expected to present a broad OH stretching band appearing at a low frequency characteristic of H-bonded OH groups, while the remaining conformers are expected to give rise to narrow OH stretching bands originating in their non-hydrogen-bonded hydroxyl group, which should be observed above 3600 cm^–1^. The broadband of conformer **I** is in fact observed experimentally between 3240 and 3100 cm^–1^, with absolute maximum at 3132 cm^–1^, while no bands are observed in the experimental spectrum above this region. This result, *per se*, clearly demonstrates the sole presence of conformer **I** in the as-deposited matrix. Nevertheless, as mentioned above, the whole spectrum represents clearly the vibrational signature of this conformer (and only of this conformer).

The proposed IR assignment of the as-deposited matrix of 5CSA in solid N_2_ is provided in Table 2. Considering the good agreement between the experimental and calculated data, the assignments were made straightforwardly. The following discussion refers only to the assignment of a few bands, which deserve here some additional comments.

**Table 2:** Assignment of the IR Spectrum of the As-Deposited 5CSA Isolated in a N_2_ Matrix (Conformer **I**)^b^

exptl ν̃	calcd ν̃	calcd *I*^IR^	symmetry	assignment (PED)
3201, 3184, 3158, **3132**, 3126, 3119	3270.7	244.8	A′	99 [ν(OH)]
3093	3060.6	0.9	A′	96 [ν_a_(CH)]
3070	3047.9	0.7	A′	97 [ν_b_(CH)]
3062	3040.2	0.8	A′	99 [ν_c_(CH)]
2886, **2860**, 2851,2845, 2835, 2772, 2762, **2740**^a^	2818.8	77.7	A′	100 [ν_al_(CH)]
1685, 1668^a^	1681.1	407.1	A′	72 [ν(C=O)]
1620	1624.4	26.3	A′	60 [ν_a_(CC)]
1584, 1582, **1580**	1579.4	45.3	A′	54 [ν_b_(CC)] + 16 [δ (OH)]
1486, 1483, 1480, **1475**	1474.1	181.6	A′	35 [δ_b_(CH)] + 21 [ν_c_(CC)]
1432, **1422**, 1416	1434.8	6.2	A′	17 [ν_e_(CC)]
1381, 1378	1389.3	45.8	A′	41 [δ_al_(CH)] + 28 [δ (OH)] + 16 ν_d_(CC)
**1342**, 1338, 1335	1368.6	66.5	A′	43 [ν_e_(CC)] + 29 [δ_al_(CH)]
1312	1321.0	43.1	A′	32 [ν_d_(CC)] + 11 [ν_e_(CC)]
1292, 1290, 1289, 1287, **1285**, 1283, **1282**, 1280	1290.5	141.4	A′	43 [ν(C–O)] + 18 [δ_b_(CH)]
1231, 1229, **1220**, 1218	1226.5	65.3	A′	37 [δ_a_(CH)]
**1185**, **1184**, **1183**, 1181, 1179, 1178, 1175	1183.1	104.1	A′	24 [ν(C1–C10] + 22 [ν_d_(CC)] + 16 [δ_c_(CH)]
1132, **1130**, 1128, 1127	1129.9	2.3	A′	45 [δ_c_(CH)] + 24 [ν_d_(CC)]
1097, **1096**, **1086**, 1085, 1084	1082.8	18.0	A′	23 [ν_c_(CC)] + 19 [ν_f_(CC)] + 18 [δ_a_(CH)]
n.obs.	1001.9	2.3	A″	72 [τ_al_(CH)] + 10 [τ(C=O)]
n.obs.	956.2	0.04	A″	112 [γ_b_(CH)]
909	901.0	19.1	A′	47 [δ(CC)] + 11 [ν(C–Cl)]
888, 887, 885, **883**, 881, 879	875.4	14.3	A″	103 [γ_a_(CH)]
**839**, **838**, 837, 834, **833**	830.3	16.2	A″	85 [γ_c_(CH)] + 17 [γ(COH)]
771, 770	768.3	14.3	A′	36 [ν_f_(CC)]
753, 752, 749, 748, 746, 744, 741, 739, 736, **734**, 731	759.5	123.1	A″	97 [τ(OH)]
**720**, **717**, 716, 714	723.3	62.1	A′	35 [δ(C=O)] + 16 [ν(C–Cl)]
n.obs.	700.0	0.1	A″	64 [τ_a_(CC)] + 33 [γ(COH)]
645, 644	640.6	25.5	A′	38 [δ_c_(CC)] + 16 [ν(C–Cl)]
549, 548, 547, **546**, **454**	541.4	9.9	A″	23 [γ(COH)] + 22 [τ_a_(CC)] + 19 [γ(C–Cl)]
458, 457	458.4	10.0	A′	61 [δ(COH)] + 18 [δ(C=O)]
437	431.8	1.2	A″	55 [τ_c_(CC)] + 33 [τ_b_(CC)] + 15 [γ(CHO)]
427	426.5	3.5	A′	47 [δ_b_(CC)] + 17 [ν(C1–C10)]
n.i.	368.6	0.5	A′	40 [ν(C–Cl)] + 33 [δ_c_(CC)]
n.i.	332.8	0.02	A″	31 [γ(C–Cl)] + 28 [γ(COH)] + 21 [τ_a_(CC)]
n.i.	294.9	6.1	A′	33 [δ(CHO)] + 27 [δ(C–Cl]
n.i.	262.2	5.4	A″	50 [τ(C=O)] + 19 [τ_al_(CH)] + 18 [γ(C–Cl)]
n.i.	202.8	4.2	A′	48 [δ(C–Cl)] + 37 [δ(CHO)]
n.i.	137.5	0.004	A″	39 [γ(CHO)] + 38 [τ_c_(CC)]
n.i.	114.8	2.8	A″	47 [τ_b_(CC)] + 24 [γ(C–Cl)]

a Fermi resonance (see the text for discussion). ν, stretching; δ, in-plane bending; γ, out-of-plane rocking; τ, torsion; al, aldehyde; n.obs., not observed; n.i., not investigated; experimental bands in bold correspond to the most intense bands in a multiplet of bands assigned to the same mode. PEDs are expressed in %, and the PED matrices lower than 15% are not included. Definition of internal coordinates is given in Table S1.

b Frequencies in cm^–1^; calculated intensities (I^IR^) in km mol^–1^; calculated frequencies scaled by 0.983 and 0.955, below and above 1800 cm^–1^, respectively.

The position and profile of the OH stretching band have already been pointed out above, and they are a clear signature of the intramolecular hydrogen bond present in conformer **I**. Still in the high-frequency region, the bands due to the CH stretching modes are observed in the 3100–3040 cm^–1^ range, in the case of the three vibrations of the CH groups of the ring, and within the 2900–2720 cm^–1^ region, for the aldehyde CH stretching mode. This latter mode, as it is usually found in aldehydes, appears for low frequencies and is involved in Fermi resonance, due to coupling with the 1st overtone of the CH aldehyde in-plane bending mode (whose fundamental gives rise to bands in the 1435–1375 cm^–1^ range). The unusually low frequency of the aldehyde CH stretching mode is well-known to result from the weakening of the C–H aldehyde bond due to the back-donation effect from the *trans* lone electron pair of the carbonyl oxygen atom.^30,31^ Note that the number of observed band-components assigned to the CH stretching aldehyde mode is larger than two in result of matrix-site splitting (as it is most of the times observed in matrix isolation IR spectra), but the components are located in two frequency regions, 2900–2820 and 2800–2720 cm^–1^ (see Table 2 and Figure 3), in agreement with the expectations. Matrix site splitting is also the reason for the other observed multicomponent bands, like, for example, that assigned to the OH stretching vibration discussed above (see also Table 2).

The C=O stretching mode in conformer **I** gives rise to a doublet of bands at 1685 and 1668 cm^–1^. This is also a Fermi doublet, resulting from the interaction of the C=O stretching fundamental with the 1st overtone of the in-phase out-of-plane CH rocking of the CH ring bonds in position *meta* and *para* to the aldehyde group, whose fundamental is observed in the 840–830 cm^–1^ region. As it will be seen in Section 4.3, this is a unique vibrational characteristic of conformer **I**, due to the fact that the carbonyl stretching appears in this form at a considerably lower frequency than in the remaining conformers because of the involvement of the carbonyl group in the intramolecular H-bond. In the remaining conformers, the carbonyl stretching shall appear at a higher frequency (above 1710 cm^–1^), and the Fermi resonance is not anymore operative, so that a single carbonyl stretching band shall be observed (as it effectively happens in the case of conformer **II**; see below).

The CCl stretching coordinate contributes to several normal modes (calculated frequencies: 901.0, 723.3, 640.6, 541.4, and 368.6 cm^–1^ see Table 2), so that the assignment of the C–Cl vibration is not straightforward. Literature data for the chlorotoluene isomers^32^ suggested the assignment of this mode to the bands observed at 1054, 1079, and 1090 cm^–1^, respectively, for *ortho-*, *meta-*, and *para*-substituted isomers, which are also similar to those found in the chloroanisoles and chlorophenetoles.^33^ For chlorobenzene, the ν(C–Cl) stretching mode was observed at 707.7 cm^–1^.^34^ Though this particular question deserves further investigation, we shall notice that the band at 909 cm^–1^, which among those predicted to have a significant contribution of the C–Cl stretching coordinate is the one appearing closer to the frequency values reported for the chlorotoluenes, chloroanisoles, and chlorophenetoles,^32,33^ is intense and shows an asymmetric profile, which is compatible with the existence of two overlapping component-bands that, after deconvolution, were shown to have their maxima at 909.3 and 908.7 cm^–1^ and an intensity ratio of approximately 3:1, as expected for molecules bearing the ^35^Cl and ^37^Cl isotopes, respectively.

Finally, the torsional mode of the hydroxyl group is observed in the 755–730 cm^–1^ range, exhibiting extensive site-splitting, with at least 11 components, and absolute maximum at 734 cm^–1^.

### 4.3 UV-Induced Rotamerization in Matrix-Isolated 5CSA

**Figure 4:**
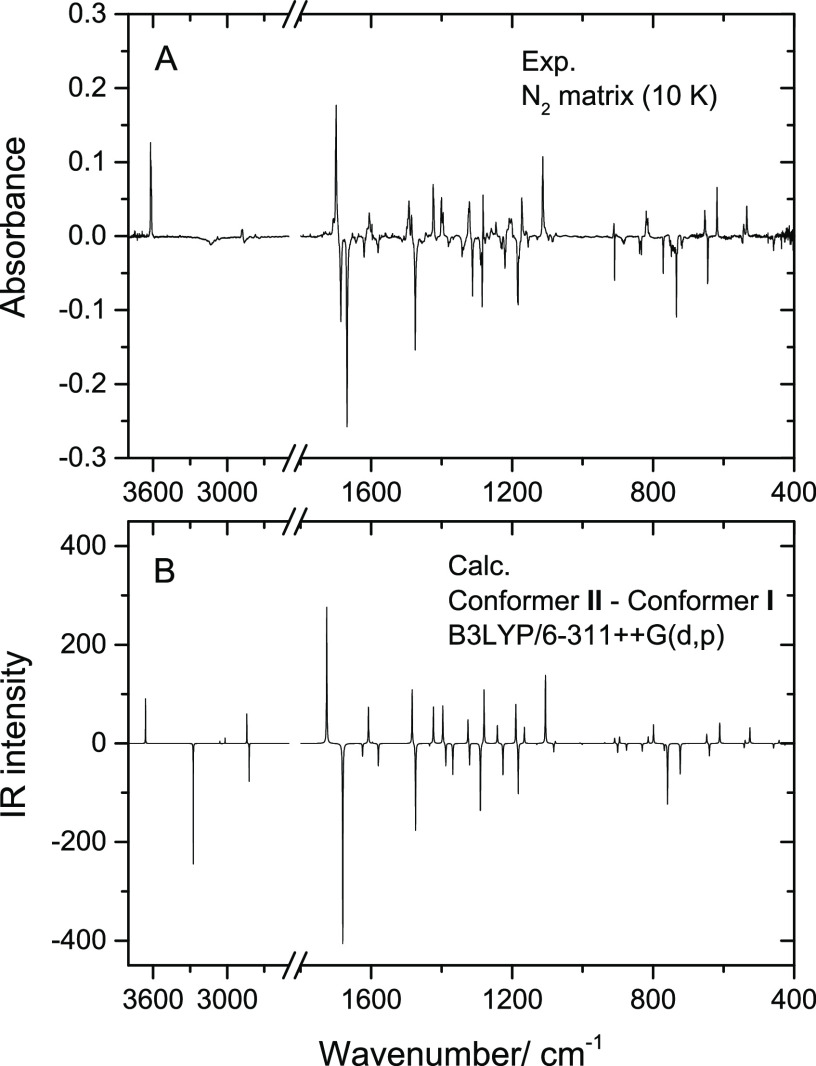
(A) Experimental IR difference spectrum (UV-irradiated matrix minus as-deposited matrix), and (B) simulated IR difference spectrum (calculated spectrum of conformer **II***minus* calculated spectrum of conformer **I**). The irradiation was performed at λ = 308 nm, for 135 min, and see Figure S2 for full-range spectra.

The matrix-isolated 5CSA was subjected to in situ UV-irradiation (λ = 308 nm) for 135 min. The UV irradiation leads to consumption of the initially deposited conformer **I** (by 35%) and emergence of new bands, which correspond to the spectrum of conformer **II**. The results are presented in Figure 4, where the difference experimental IR spectrum (irradiated matrix *minus* as-deposited matrix) is compared with the simulated **II***minus***I** IR difference spectrum built using the B3LYP/6-311++G(d,p) calculated spectra of these two conformers.

The comparison of the experimental and calculated data leaves no doubts regarding the nature of the photoproduced conformer. The obtained results are in line with those reported by Lapinski et al. for salicylaldehyde,^9^ where the intramolecularly H-bonded most-stable conformer of that compound could also be successfully converted into a conformer identical to form **II** of 5CSA by broadband UV irradiation (λ > 335 nm) of the compound isolated in an argon matrix. In the case of salicylaldehyde subjected to broadband UV irradiation,^9^ the observed rotamerization reaction was found to be accompanied by minor production of ketene species, which was identified by its characteristic intense absorption in the 2140–2115 cm^–1^ spectral region. Very interestingly, no evidence of any other photoreaction, besides the **I** → **II** rotamerization, was observed in the present study, for 5CSA isolated in solid N_2_.

Assignment of the IR spectrum of conformer **II** is provided in Table 3. As for conformer **I**, the excellent agreement between the calculated and experimental data allowed the assignment of the spectrum of form **II** to be made easily. The following observations shall be highlighted:

(i) As expected, the OH stretching band of conformer **II** exhibits a narrow profile and characteristic frequency (absolute maximum at 3620 cm^–1^; i.e., shifted by 488 cm^–1^ regarding the maximum of the broadband due to the OH stretching mode of conformer **I**) of a non-hydrogen-bonded OH group. The OH stretching band of **II** is, however, as most of the other observed bands for both conformers **I** and **II**, matrix-site split, as indicated in Table 3.
(ii) The carbonyl stretching vibration gives rise, in conformer **II**, to a band at 1699 cm^–1^, which is shifted to higher frequencies regarding its position in conformer **I**, as expected. In this case, the band does not show any evidence of involvement of the C=O stretching mode in any Fermi resonance interaction, contrarily to what happens in the case of conformer **I**. Indeed, the fact that the C=O stretching mode in conformer **II** absorbs at a considerably higher frequency than in conformer **I**, together with the fact that the 1st overtone of the in-phase out-of-plane CH rocking of the CH ring bonds in position *meta* and *para* to the aldehyde group (which is the Fermi resonance interacting overtone in conformer **I**) absorbs at a lower frequency in conformer **II** (the fundamental is observed in the 820–810 cm^–1^) compared to conformer **I** (fundamental: 840–830 cm^–1^ region), precludes the Fermi interaction to take place;
(iii) Like for conformer **I**, also in conformer **II**, the aldehyde CH stretching mode is observed at a low frequency and is involved in Fermi resonance with the 1st overtone of the CH aldehyde in-plane bending mode. In this case, the effect of the matrix-splitting is not so pronounced as for form **I**, and the two bands of the Fermi doublet are even more clearly visible, with the absolute maxima being observed at 2878 and 2772 cm^–1^ (see Table 3).

(iv) The calculated band of conformer **II** at 895.8 cm^–1^ is the counterpart of that predicted at 901.0 cm^–1^ for conformer I. However, for conformer **II**, the band is assigned to the experimental feature at 906 cm^–1^, which is partially overlapped with the band of conformer **I**. This fact precludes a suitable curve fitting, so that possible contributions due to the molecules bearing the two isotopes of chlorine could not be identified in this case.
(v) It shall also be noticed that the torsional mode of the hydroxyl group in conformer **II** is predicted to be shifted to below 300 cm^–1^, that is, the typical range of frequencies for a non-hydrogen-bonded torsion OH phenolic group.^35^ This frequency is outside the accessible frequency range of our instrumentation and could not therefore be assigned.

**Table 3:** Assignment of the IR Spectrum of the Photoproduced Conformer **II** of 5CSA Isolated in a N_2_ Matrix^a^

exptl ν̃	calcd ν̃	calcd *I*^IR^	symmetry	assignment (PED)
**3620**, 3618, 3616, 3614, 3612	3660.9	90.5	A′	100 [ν(OH)]
3062	3059.1	4.7	A′	100 [ν_c_(CH)]
3062	3056.7	0.5	A′	62 [ν_a_(CH)] + 30 [ν_b_(CH)]
3040, 3029	3016.1	11.0	A′	66 [ν_b_(CH)] + 33 [ν_a_(CH)]
2883, **2878**, 2776, **2772**, 2768^b^	2841.5	60.6	A′	100 [ν_al_(CH)]
1700, **1699**	1725.4	280.1	A′	88 [ν(C=O)]
1605	1607.6	74.1	A′	65 [ν_a_(CC)]
1597	1596.5	2.2	A′	64 [ν_b_(CC)]
1498, 1496, **1493**, 1491, 1485	1484.0	110.1	A′	39 [δ_b_(CH)] + 19 [ν_c_(CC)] + 15 [ν_d_(CC)]
1424, 1423	1423.6	76.3	A′	25 [ν_e_(CC)] + 21[δ_al_(CH)] + 16 [ν_c_(CC)]
1401, **1400**, 1397, 1396	1396.5	78.5	A′	63[δ_al_(CH)]
1324, 1323, **1321**, 1320	1325.4	49.6	A′	35 [ν_e_(CC)] + 28 [ν_d_(CC)] + 20 [δ(OH)]
1283, 1281	1279.7	109.9	A′	38 [δ_b_(CH)] + 20 [δ_a_(CH)] + 18 [ν_e_(CC)] + 15 [ν(C–O)]
1246, 1244	1241.9	37.3	A′	32 [ν(C–O)] + 15 [δ_a_(CH)]
1208, 1207, 1202	1189.8	81.8	A′	28 [ν(C1–C10)] + 24 [δ(OH)] + 12 [ν_f_(CC)]
**1172**, 1170, 1168	1165.5	34.1	A′	53 [δ_c_(CH)] + 17 [ν_e_(CC)] + 17 [δ(OH)]
1113	1106.2	138.7	A′	22 [δ_a_(CC)] + 20 [ν_d_(CC)] + 16 [ν_c_(CC)]
1076	1077.8	4.4	A′	28 [ν_d_(CC)] + 20 [δ_a_(CH)]
n.obs.	1007.7	0.9	A″	66 [τ_al_(CH)] + 22 [τ(C=O)]
930	936.9	1.4	A″	83 [γ_b_(CH)] + 24 [γ_a_(CH)]
913, 912, **911**	909.0	11.1	A″	78 [γ_a_(CH)] + 28 [γ_b_(CH)]
906	895.8	13.4	A′	26 [δ_a_(CC)] + 18 [ν(C1–C10)] + 16 [ν(C–Cl)]
820, **819**	814.1	13.9	A′	32 [ν_f_(CC)] + 20 [ν(C–O)] + 16 [δ(C=O)]
818, 817, **815**, 812, 810	798.4	38.6	A″	87 [γ_c_(CH)] + 13 [γ(COH)]
n.obs.	688.7	0.1	A″	73 [τ_a_(CC)] + 27 [γ(COH)]
653, 652	648.3	19.5	A′	40 [δ_c_(CC)] + 16 [ν(C–Cl)]
619	611.5	41.8	A′	34 [δ(C=O)] + 18 [δ_b_(CC)] + 15 [ν_e_(CC)]
543, 542	538.9	7.2	A″	24 [γ(COH)] + 21[γ(C–Cl)] + 15 [τ_a_(CC)]
535	525.7	32.1	A′	38 [δ(COH)] + 17 [ν(C1–C10)]
446	443.2	6.4	A″	44 [τ_c_(CC)] + 33 [τ_b_(CC)] + 19 [γ(CHO)]
n.i.	378.6	0.1	A′	31 [δ_c_(CC)] + 29 [ν(C–Cl)]
n.i.	361.3	5.9	A′	35 [δ_b_(CC)]
n.i.	335.7	1.9	A″	33 [γ(C–Cl)] + 22 [γ(COH)] + 17 [τ_a_(CC)]
n.i.	285.5	90.4	A″	95 [τ(OH)]
n.i.	277.1	1.5	A′	43 [δ(C–Cl)] + 25 [δ(COH)] + 20 [δ(CHO)]
n.i.	174.6	23.6	A″	34 [γ(CHO)] + 19 [τ_c_(CC)] + 15 [γ(C–Cl)]
n.i.	164.5	3.0	A′	52 [δ(CHO)] + 26 [δ(C–Cl)]
n.i.	128.7	5.8	A″	38 [τ(C=O)] + 22 [τ_b_(CC)]
n.i.	103.8	0.8	A″	33 [τ(C=O)] + 31 [τ_b_(CC)] + 23 [τ_c_(CC)]

a Frequencies in cm^–1^; calculated intensities (I^IR^) in km mol^–1^; calculated frequencies scaled by 0.983 and 0.955, below and above 1800 cm^–1^, respectively.

b Fermi resonance (see the text for discussion). ν, stretching; δ, in-plane bending; γ, out-of-plane rocking; τ, torsion; o, m, p: CH group *ortho*, *meta*, and *para* to the aldehyde substituent; al, aldehyde; n.obs., not observed; n.i., not investigated; experimental bands in bold correspond to the most intense bands in a multiplet of bands assigned to the same mode. PEDs are expressed in %, and the PED matrices lower than 15% are not included. Definition of internal coordinates is given in Table S1.

**Figure 5:**
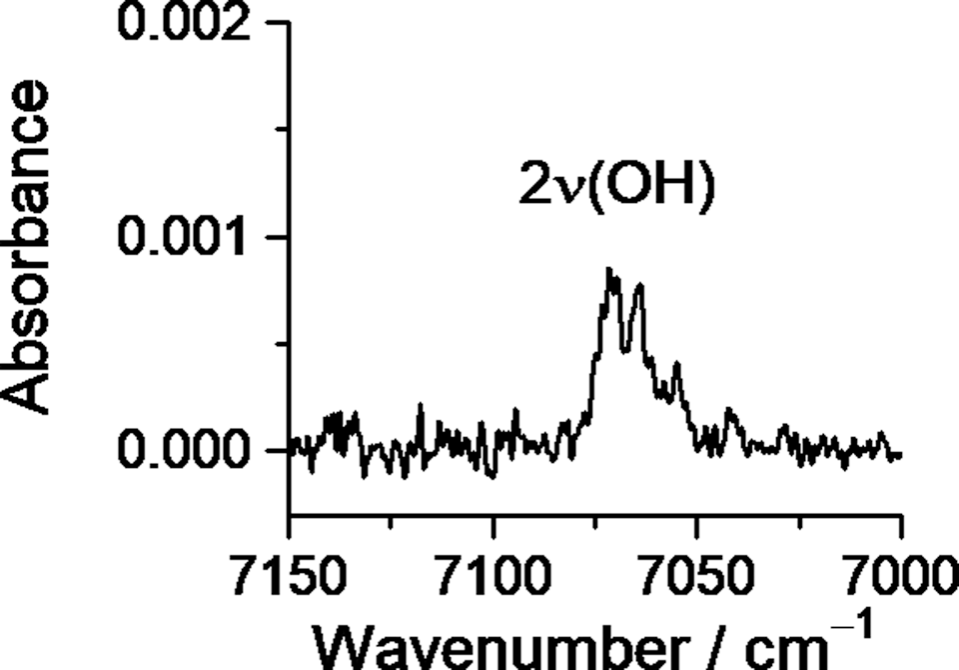
Experimental near-IR spectrum of 5CSA isolated in a N_2_ matrix, at 10 K subsequent to UV-induced generation of conformer **II**.

The fact that the OH stretching band of conformer **II** is found narrow and at high frequencies encouraged the recording of its near-IR spectrum to assign the 2ν(OH) mode, see Figure 5. Excitations of specific vibrational modes, namely, 2ν(OH), by tunable and narrowband OPO:LASERS have been successfully employed to induced conformational isomerizations^36,37^ and as of lately also chemical reactions involving bond-breaking and bond-forming.^29,38,39^ It was therefore envisaged that upon vibrational excitation of the 2ν(OH) mode of conformer **II**, the rotamerization of the OH group around its C–O bond could take place to generate conformer **III**.

The first OH stretching overtone of conformer **II** also exhibits site-splitting, as found for the fundamental transition, with the strongest components found at 7071 and 7064 cm^–1^. Irradiations of matrix-isolated **II** performed at those wavenumbers did not, however, lead to changes in the IR spectra of 5CSA. As discussed in Section 4.1, the barrier for internal rotation of the OH group in conformer **III** amounts to 11.0 kJ mol^–1^, see Figure 2, and involves mainly the displacement of the light hydroxyl hydrogen atom, which could justify a fast decay of this conformer back to conformer **II** by H-atom tunneling. IRC computations performed for the reactions depicted in Figure 2 allowed quantum tunneling rate constants to be predicted (Table 4 and Figure S1) and justify this hypothesis.

**Table 4:** Parameters of the Potential Energy Barriers along the Intrinsic Reaction Coordinate for the Isomerization Reactions Depicted in Figure 2 and Reaction Rates via H-Atom Tunneling from the Vibrational Ground State

		barrier^a^		rate constants^b^	
reaction	fragment	width (bohr)	height (kJ mol^–1^)	*k* (s^–1^)	*t*_1/2_ (s)
**III** → **I**	CHO^c^	5.27	29.20	4.18 × 10^–11^	1.66 × 10^10^
**IV** → **II**	CHO^c^	5.18	20.20	4.4 × 10^–7^	1.57 × 10^6^
**IV** → **I**	OH	2.11	11.23	1.84 × 10^7^	3.77 × 10^–8^
**III** → **II**	OH	2.61	8.64	8.08 × 10^6^	8.58 × 10^–8^

a Computed at the DFT(B3LYP)/6-311++G(d,p) level of theory.

b Estimated using the Wentzel–Kramers–Brillouin (WKB) formalism.

c The displacement of the heavy CHO group was assumed to have a reduced mass of 1 amu (H-atom).

The analysis of the data reported in Table 4 indicates two distinct scenarios. The first concerns reactions where the heavy CHO fragment isomerizes. For those, the barriers are wide (>5 bohr) and the rate constants negligible (<10^–7^ s^–1^). Note that for the sake of simplification, the rate constants were predicted assuming a reduced mass of 1 amu along the reaction coordinate, and for that reason, one would expect the constants in practice to be even lower due to the exponential dependence of quantum tunneling with the reduced mass along the reaction coordinate. On the other hand, the energy profiles for the reactions that involve the displacement of the hydrogen atom of the hydroxyl group are thinner and should occur extremely fast (rate constants in the range of 10^6^ or 10^7^ s^–1^, which translates to half-life times in the nanosecond timescale). Hence, conformers **III** and **IV** should be considered fleeting species, whose experimental detection by steady-state spectroscopic methods does not seem to be achievable, even under low-temperature matrix isolation conditions. In fact, it is likely that the formation of conformer **II** occurs through two steps—the initial UV-induced aldehyde rotamerization of conformer **I** (**I** → **III**), followed by a fast OH rotamerization (**III** → **II**) by quantum tunneling. The fleeting nature of molecules due to quantum tunneling is not unprecedented^40^ and was discussed in great detailed by Kozuch and co-workers, who titled it *tunneling instability*.^41^

A final note shall be made here regarding the fact that conformer **II** was found to be stable in the low-temperature matrix, with no evidence found of its spontaneous back-conversion to conformer **I** after its production. As discussed in Section 4.1, the **II** → **I** rotamerization always involves a substantial movement of the aldehyde oxygen atom, and the isomerization barrier is certainly wide enough to prevent spontaneous conversion of **II** into **I** via quantum mechanical tunneling in the cryogenic matrix. This result agrees also with the previous observations on salicylaldehyde, where the analogous conformer of **II** was also found to be stable in a low-temperature argon matrix, once photogenerated in situ.^9^

### 4.4 Low-Temperature IR Spectra of Amorphous and Crystalline 5CSA

**Figure 6:**
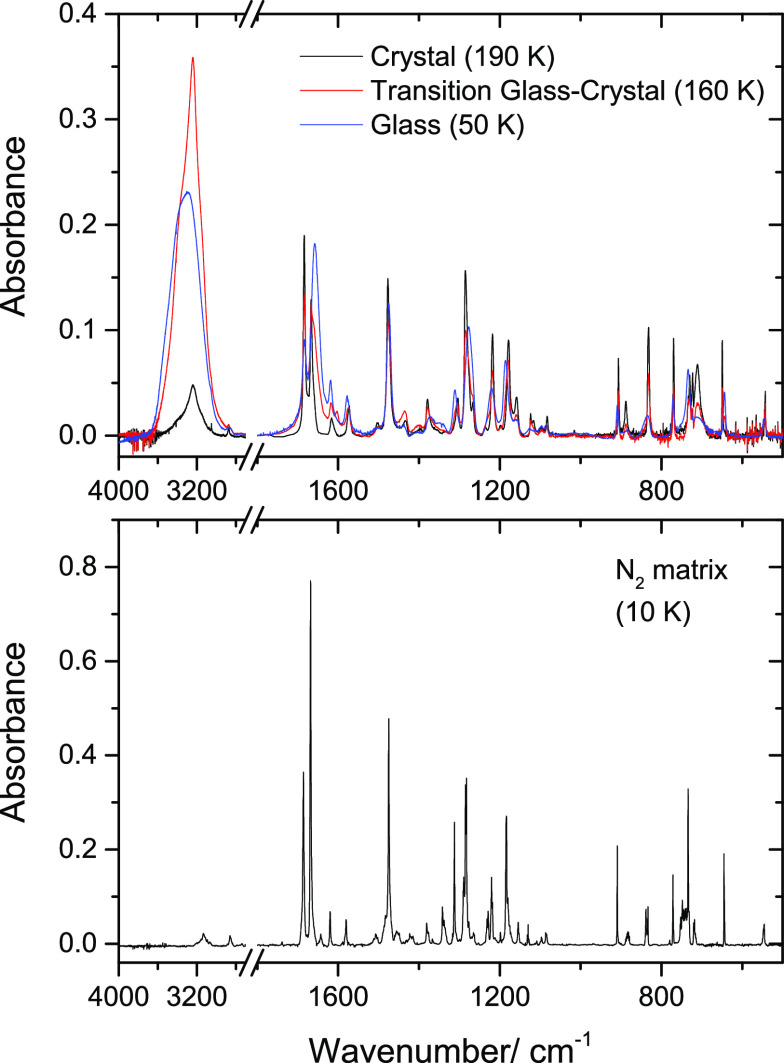
Experimental IR spectra of neat 5CSA at different temperatures showing the transition from the glassy state to the crystalline phase of the compound (*top*). The spectrum of the compound in a solid N_2_ matrix is shown in the *bottom* panel, for comparison.

An amorphous sample of neat 5CSA was prepared from a previously deposited N_2_ matrix of the compound, by evaporation of the N_2_ host material and molecular diffusion upon warming. At 50 K, a thin layer of the neat glassy 5CSA exhibiting good optical properties was obtained. The spectrum of the glass material is shown in Figure 6. The spectrum has the typical profile of an amorphous state, with several bands exhibiting a broad profile, as for example the OH stretching band, observed as a wide intense feature between 3700 and 2900 cm^–1^ (with absolute maximum at 3300 cm^–1^), the C=O stretching Fermi doublet, observed at 1683 and 1658 cm^–1^, and that assigned to the OH torsion, which spread between ca. 730 and 680 cm^–1^, with maximum at 708 cm^–1^. The broadened bands are all ascribed to vibrations of the groups, which participate in the most relevant intermolecular interactions, i.e., intermolecular hydrogen bonds, where the carbonyl group acts as the acceptor and the hydroxyl group can act both as the acceptor and donor.

It is interesting to note that the O–H^···^O= intramolecular hydrogen bond is preserved in the glassy state, which implies that the intermolecular hydrogen bonds are of the bifurcated type; i.e., the OH groups participate simultaneously as donors in the intramolecular bond characteristic of conformer **I** and in an intermolecular H-bond with another molecule. Supporting this conclusion is the observation that the spectrum of the glassy state resembles very much that of conformer **I** (see Figure 6), as well as the fact that this situation happens in the crystal structure of 5CSA.^16,17^

A straightforward assignment of the spectrum of the amorphous 5CSA could be given (Table 5) due to the general similarity of the spectra of this condensed phase and that observed for the matrix-isolated compound for the vibrations that are not localized in the hydroxyl and carbonyl moieties, since those are directly involved in the intermolecular interactions.

**Table 5:** Assignment of the IR Spectra of 5CSA in the Glassy State (at 50 K) and Crystalline Phase (190 K)^a^

glassy state (50 K)	crystal (190 K)	assignment
3300	3240	99 [ν(OH)]
n.obs	3080	96 [ν_a_(CH)]
n.obs.	3080	97 [ν_b_(CH)]
3050	3050	99 [ν_c_(CH)]
2875, 2756^b^	2875, 2750^b^	100 [ν_al_(CH)]
1683, 1658^b^	1683, 1667^b^	72 [ν(C=O)]
1618	1616	60 [ν_a_(CC)]
1577	1572	54 [ν_b_(CC)] + 16 [δ (OH)]
1475	1477	35 [δ_b_(CH)] + 21 [ν_c_(CC)]
1437	1435	17 [ν_e_(CC)]
1374	1379	41 [δ_al_(CH)] + 28 [δ (OH)] + 16 ν_d_(CC)
1349	1335	43 [ν_e_(CC)] + 29 [δ_al_(CH)]
1311	1304	32 [ν_d_(CC)] + 11 [ν_e_(CC)]
1277	1285, 1265	43 [ν(C–O)] + 18 [δ_b_(CH)]
1220	1236, 1218	37 [δ_a_(CH)]
1186, 1162	1178, 1158	24 [ν(C1–C10] + 22 [ν_d_(CC)] + 16 [δ_c_(CH)]
1127, 1113	1123, 1117	45 [δ_c_(CH)] + 24 [ν_d_(CC)]
1095, 1086	1097, 1083	23 [ν_c_(CC)] + 19 [ν_f_(CC)] + 18 [δ_a_(CH)]
1013	1015	72 [τ_al_(CH)] + 10 [τ(C=O)]
n.obs	954	112 [γ_b_(CH)]
909	906	47 [δ(CC)] + 11 [ν(C–Cl)]
883	887	103 [γ_a_(CH)]
834	832	85 [γ_c_(CH)] + 17 [γ(COH)]
770	770	36 [ν_f_(CC)]
708	710	97 [τ(OH)]
733	730, 722	35 [δ(C=O)] + 16 [ν(C–Cl)]
n.obs.	n.obs.	64 [τ_a_(CC)] + 33 [γ(COH)]
644	649	38 [δ_c_(CC)] + 16 [ν(C–Cl)]
545	543	23 [γ(COH)] + 22 [τ_a_(CC)] + 19 [γ(C–Cl)]
461	456	61 [δ(COH)] + 18 [δ(C=O)]
438	437	55 [τ_c_(CC)] + 33 [τ_b_(CC)] + 15 [γ(CHO)]
425	425	47 [δ_b_(CC)] + 17 [ν(C1–C10)]

a Frequencies in cm^–1^.

b Fermi resonance (see the text for discussion). ν, stretching; δ, in-plane bending; γ, out-of-plane rocking; τ, torsion; o, m, p: CH group *ortho*, *meta*, and *para* to the aldehyde substituent; al, aldehyde; n.obs., not observed. The assignment is given considering the calculated PEDs (expressed in %) for conformer **I** isolated in vacuo. PED matrices lower than 15% are not included. Definition of internal coordinates is given in Table S1.

Increasing of the temperature led to crystallization of the compound, which started at around 150 K and was complete at 190 K. Figure 6 shows also the spectrum of the crystalline phase (at 190 K), which exhibits characteristic narrow bands (compared to that of an amorphous phase), as well as the spectrum obtained at 160 K, obtained while the crystallization was proceeding and that shows a mixed signature of the amorphous and crystalline phases. The assignment of the spectrum of the crystal is provided in Table 5, where it can be compared with that of the glassy state.

There are some interesting observations that deserve additional comments. The first one is the large difference in the broadness, frequency, and intensity of the OH stretching band in the amorphous state compared to the crystalline phase (see Figure 6). In the spectrum of the amorphous phase, the OH band is both much broader and intense than in the spectrum of the crystalline phase, and it is observed at a higher frequency. The increased broadness is easily attributed to the high degree of molecular disorder in the glassy state, which leads to a dispersion of the energy levels associated with this vibration due to the simultaneous presence of intermolecular H-bonds of different strengths. On the other hand, the increase of intensity in going from the crystal to the glassy state must be related to a larger bond-dipole moment in the latter, which probably takes place due to a closer packing of the molecules in the disordered amorphous phase, compared to the periodic arrangement in the crystal. The staking-type interactions present in the crystal, where the molecules are aligned so that the OH groups of neighbor molecules superimposed (see Figure S3 in the Supporting Information), are certainly relevant in decreasing the OH bond dipole, thus reducing the IR intensity of the associated stretching vibration. On the other hand, in the glassy state, the antiparallel alignment of the OH groups appears to be energetically more favorable and shall predominate, increasing the polarization of the OH bond. The relevance of intermolecular interactions in determining the intensity of the O–H stretching band in the different phases studied is also confirmed by the much higher intensity (at least, by an order of magnitude) of the band in the spectra of the neat compound (both in the glassy or crystalline phase) than in the spectrum of the isolated 5CSA molecule in the N_2_ matrix. Regarding the frequency of the OH stretching mode, it shall be noticed that it follows the order: glass (3300 cm^–1^) > crystal (3240 cm^–1^) > > isolated molecule (3132 cm^–1^), which is an indication that the intramolecular H-bond is considerably stronger alone in the isolated molecule situation, compared to the bifurcated H-bond present in the neat phases.

The last conclusion is reinforced by the analysis of the OH torsion spectral region. It is well-known that a stronger H-bond correlated with a lower OH stretching frequency and a higher OH torsion frequency.^42,43^ Accordingly, the frequency of the OH torsion follows the order: glass (708 cm^–1^) < crystal (710 cm^–1^) < < isolated molecule (753–751 cm^–1^; site split band). Note that the OH torsion band is also very much broader in the spectrum of the amorphous state of 5CSA than in that of the crystalline phase, an observation that has the same explanation as that provided above for the relative broadness of the OH stretching bands in the two spectra.

Another fact to highlight is the appearance of the carbonyl stretching Fermi doublet characteristic of conformer **I** in the spectra of both the crystal and amorphous states, which doubtlessly proves that the intramolecular H-bond is kept in both cases. In the crystalline state, this has been verified experimentally in the previously reported single-crystal X-ray diffraction studies,^16,17^ as it was already mentioned above.

Finally, it can be noticed that, while the CCl stretching band experiences the general broadening in the amorphous compound, interestingly, its frequency is the same as observed for the matrix-isolated molecules of 5CSA (909 cm^–1^ in both cases), which indicates the relative insensitivity of this vibration to the intermolecular environment. Nevertheless, the frequency of this mode decreases slightly (to 906 cm^–1^) in the neat crystalline phase, much probably due to the intermolecular contact between the chlorine atoms and the *para* CH bond (*d*_H···Cl_ = 3.141 Å)^16^ in the crystal (see Figure S2, in the Supporting Information), which is close to the sum of the van der Waals radii of Cl and H atoms (1.75 + 1.2 = 2.95 Å). This close contact in the crystal can be expected to pull electrons out of the C–Cl bond, thus reducing the corresponding CCl stretching force constant and, consequently, the associated vibrational frequency, as observed.

## 5 Conclusions

In this study, monomers of 5-chlorosalicylaldehyde were isolated in solid N_2_, at 10 K, and investigated structurally and vibrationally, by IR spectroscopy, complemented by DFT(B3LYP)/6-311++G(d,p) calculations. The calculations revealed the existence of four different conformers of the compound, whose structures, relative energies, and barriers of interconversion were obtained. According to the theoretical data, the intramolecularly hydrogen-bonded conformer **I** was found to be considerably more stable than the remaining conformers (**II**–**IV**), which do not present any intramolecular hydrogen bond and have negligible predicted populations in the room-temperature gas-phase equilibrium. The consideration of the structures of the higher-energy conformers and the potential energy barriers for conformational isomerization allowed us to conclude that conformer **II** had the conditions to be stable in a low-temperature matrix, if it could be produced in situ by any means, while conformers **III** and **IV** should be fleeting species that decay spontaneously to **II** and **I**, respectively, by quantum mechanical tunneling.

As predicted by the calculations, in the as-deposited low-temperature (10 K) N_2_ matrices of the compound, only the most stable conformer **I** was observed to be present. This conformer was successfully converted into conformer **II** (where both aldehyde and hydroxyl groups are rotated by 180° relative to their position in conformer **I**), by narrowband UV (λ = 308 nm) in situ irradiation of the as-deposited N_2_ matrix of 5CSA. Very interestingly, under the experimental conditions used in the present investigation, the observed UV-induced **I** → **II** rotamerization was found to take place in an exclusive basis, with no other photochemical processes being observed.

The compound was also investigated in its low-temperature amorphous state and in its crystalline state, at 50 and 190 K, respectively, by IR spectroscopy.

The IR spectra of both matrix-isolated conformers (the initially deposited conformer **I** and the photoproduced conformer **II**), as well as those of the neat amorphous and crystalline phases of 5CSA, were assigned and interpreted in comparative terms, allowing us to elucidate structurally and vibrationally relevant effects of the main intra- and intermolecular interactions operating in the different studied phases.

This study was the first structural, vibrational, and photochemical investigation on 5CSA (besides the previously reported X-ray studies of the crystalline phase of the compound).^16,17^

## Supporting Information Available

Figure S1 showing the IRC profiles for the conformational isomerizations in 5CSA; Figure S2 showing the full range of the spectra resulting from the photochemical experiments (expansion of Figure 4); Figure S3 showing the molecular packing of the 5CSA molecules in the crystalline phase along the crystallographic *a*, *b*, and *c* axes; Table S1 describing the definition of the internal coordinates used in the normal mode analysis for conformers **I** and **II**; Table S2 providing the Cartesian coordinates for the B3LYP/6-311++G(d,p) calculated optimized geometries of the 5CSA conformers (PDF)
