# Supplementary material for: Low-Temperature Infrared Spectra and Ultraviolet-Induced Rotamerization of 5-Chlorosalicylaldehyde
Source: J Phys Chem A. 2022 Jul 29;126(31):5148–59. doi: 10.1021/acs.jpca.2c03685 (PMC9778744; doi:10.1021/acs.jpca.2c03685)

Low-Temperature Infrared Spectra and UV-Induced Rotamerization of 5-Chlorosalicylaldehyde

Anna Luiza B. Brito,[1,*] José P. L. Roque,[1] İsa Sıdır,[1,2] and Rui Fausto[1]

[1]*CQC-IMS, Department of Chemistry, University of Coimbra, 3004-535 Coimbra, Portugal*

[2]*Department of Physics, Bitlis Eren University, 13000 Bitlis, Turkey*

Supporting Information

Index

Figure S1. IRC profiles for the conformational isomerizations in 5CSA. **S2**

Figure S2. Experimental IR spectrum of 5CSA isolated in an N_2 matrix, at 10 K (A), and simulated IR spectrum of conformer **I** of the molecule, built using the B3LYP/6-311++G(d,p) calculated vibrational data (expansion of Figure 4 showing the full spectral range) **S3**

Figure S3. 5CSA crystal packing views along the *a*, *b* and *c* crystallographic axes. **S4**

Table S1. Definition of the internal coordinates for the conformers of 5CSA. **S5**

Table S2. Cartesian coordinates for the conformers of 5CSA. **S6**

* Corresponding author: anna.brito@qui.uc.pt (A.L.B.B.)

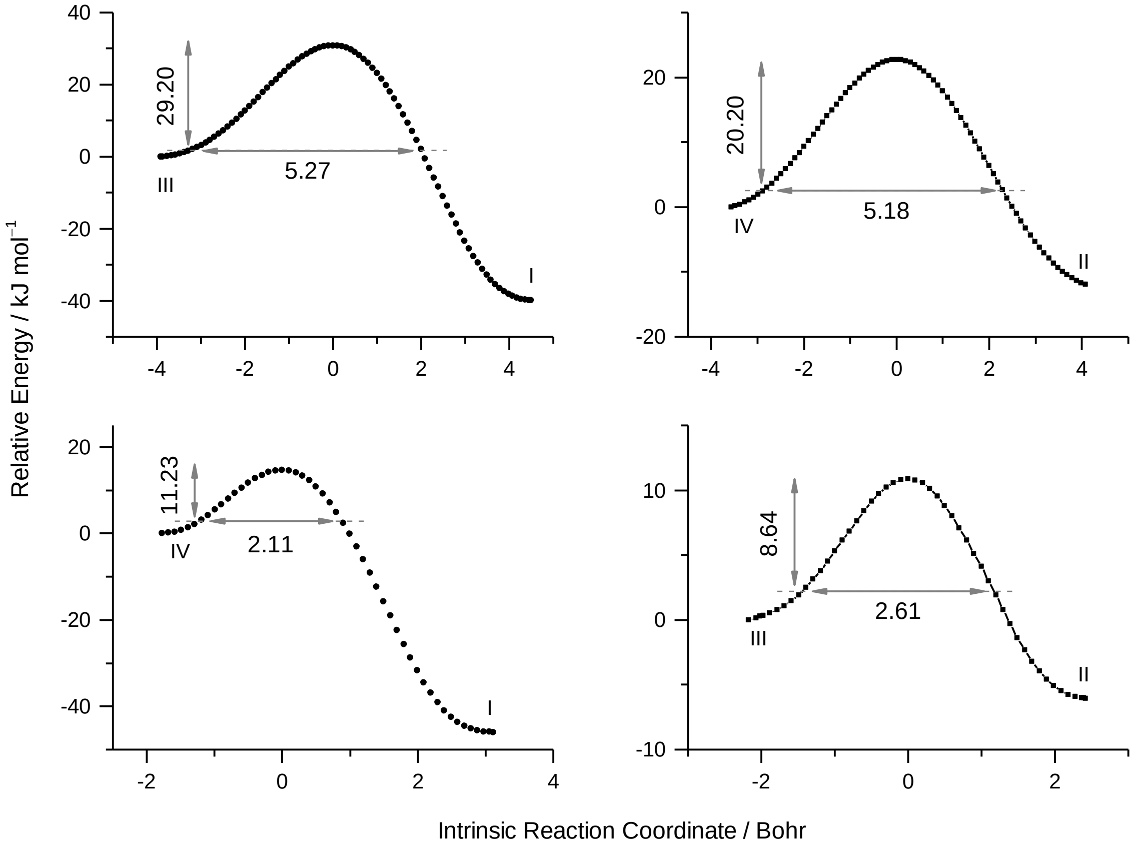

Figure S1. IRC profiles computed at the DFT(B3LYP)/6-311++G(d,p) level of theory for the conformational isomerizations in 5CSA. The horizontal dashed lines (grey) represent the zero-point vibrational energy (ZPVE) correction of the high-energy conformer relative to the corresponding transition state. The relative zero of energy for each frame was set to be the computed electronic energy of the higher energy conformer.

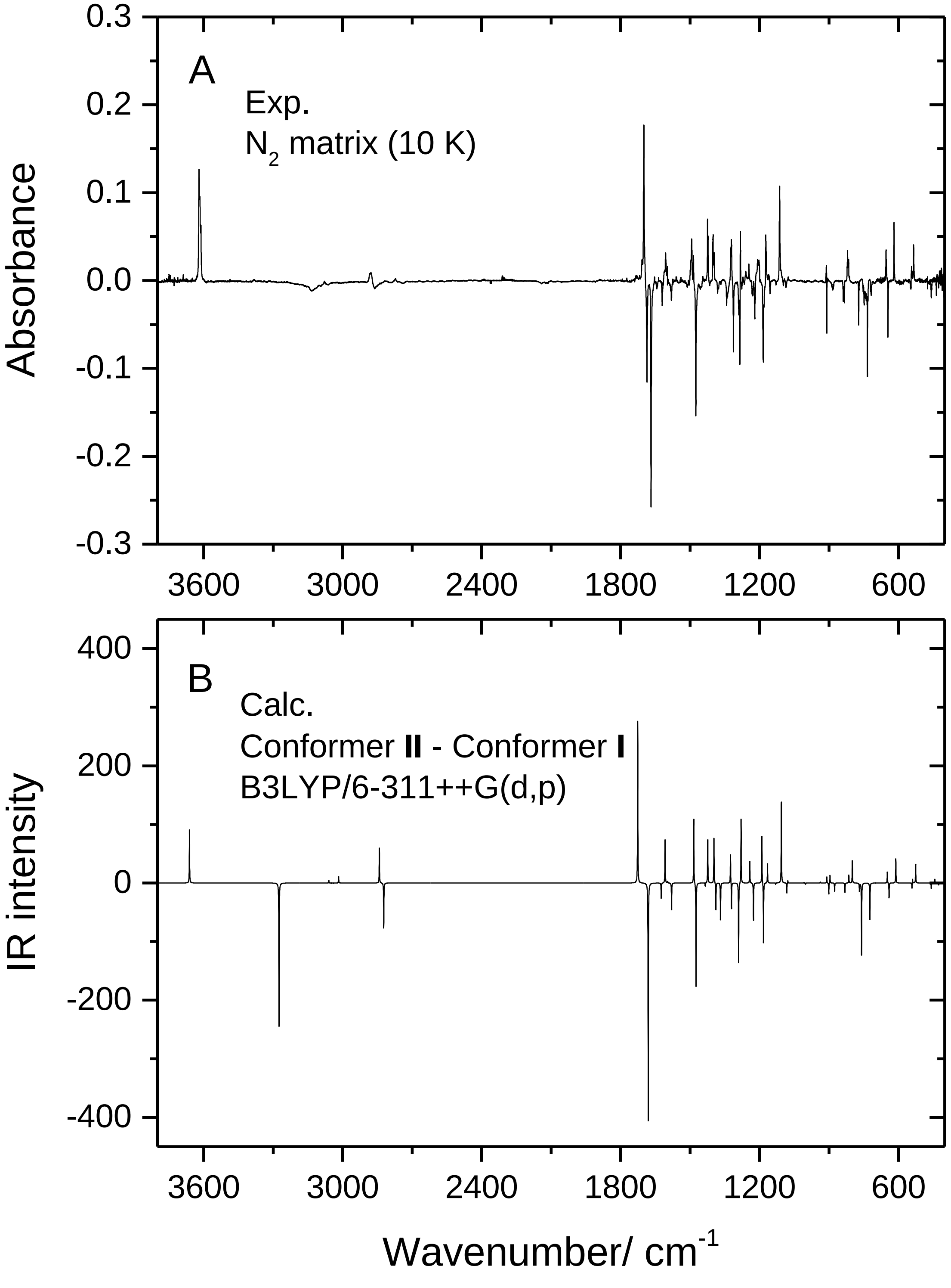

Figure S2. Experimental IR spectrum of 5CSA isolated in an N_2 matrix, at 10 K (A), and simulated IR spectrum of conformer **I** of the molecule, built using the B3LYP/6-311++G(d,p) calculated vibrational data (expansion of Figure 4 showing the full spectral range).

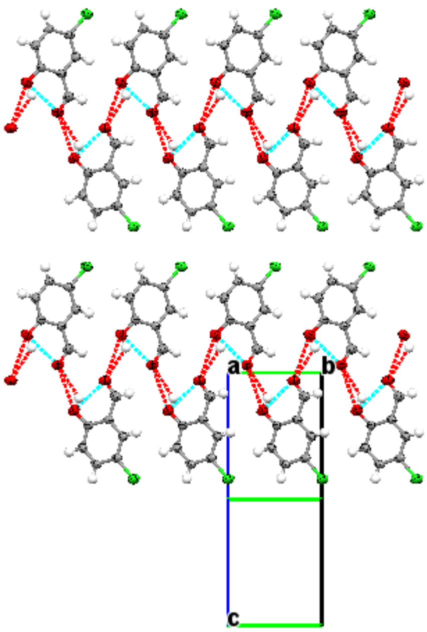

A

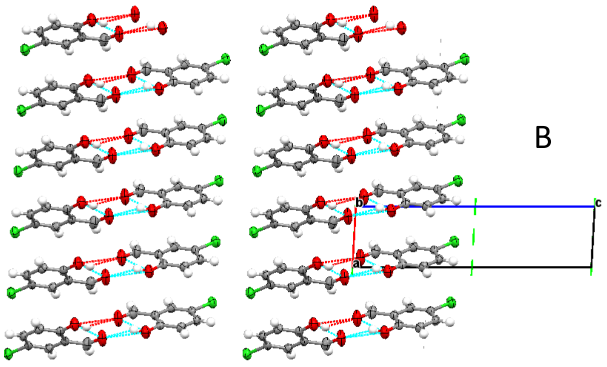

B

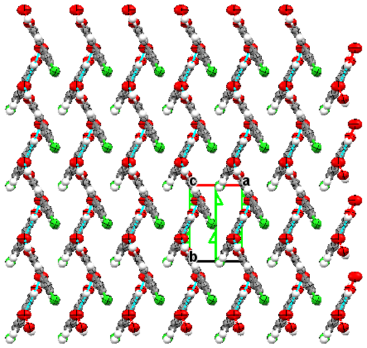

C

Figure S3. 5CSA crystal packing views along the *a*, *b* and *c* crystallographic axes (from CIF CCDC 877659.[16]

Table S1. Definition of internal coordinates used in the normal mode analysis of **I** and **II**.[a]
Definition of internal coordinates used in the normal mode analysis of **I** and **II**.[a]

Coordinate	Definition	Approximate description
	A' symmetry	
S_1	$r_{13,14}$	ν(OH)
S_2	$(2^{-1/2})(r_{3,7} + r_{5,8})$	ν_a(CH)
S_3	$(2^{-1/2})(r_{3,7} - r_{5,8})$	ν_b(CH)
S_4	$r_{6,9}$	ν_c(CH)
S_5	$r_{10,11}$	ν_{al}(CH)
S_6	$r_{10,12}$	ν(C=O)
S_7	$(12^{-1/2})(2r_{3,2} - r_{2,1} - r_{1,6} + 2r_{6,5} - r_{5,4} - r_{4,3})$	ν_a(CC)
S_8	$(4^{-1/2})(r_{2,1} - r_{1,6} + r_{5,4} - r_{4,3})$	ν_b(CC)
S_9	$(4^{-1/2})(r_{2,1} + r_{1,6} - r_{5,4} - r_{4,3})$	ν_c(CC)
S_{10}	$(12^{-1/2})(2r_{3,2} - r_{2,1} + r_{1,6} - 2r_{6,5} + r_{5,4} - r_{4,3})$	ν_d(CC)
S_{11}	$(6^{-1/2})(r_{3,2} - r_{2,1} + r_{1,6} - r_{6,5} + r_{5,4} - r_{4,3})$	ν_e(CC)
S_{12}	$(6^{-1/2})(r_{3,2} + r_{2,1} + r_{1,6} + r_{6,5} + r_{5,4} + r_{4,3})$	ν_f(CC)
S_{13}	$r_{1,10}$	ν(C1-C10)
S_{14}	$r_{2,13}$	ν(C-O)
S_{15}	$r_{5,15}$	ν(C-Cl)
S_{16}	$(2^{-1/2})(\beta_{1,9,6} - \beta_{5,9,6})$	δ_a(CH)
S_{17}	$(2^{-1/2})(\beta_{4,15,5} - \beta_{6,15,5})$	δ(C-Cl)
S_{18}	$(4^{-1/2})(\beta_{5,8,4} - \beta_{3,8,4} + \beta_{4,7,3} - \beta_{2,7,3})$	δ_b(CH)
S_{19}	$(4^{-1/2})(\beta_{5,8,4} - \beta_{3,8,4} - \beta_{4,7,3} + \beta_{2,7,3})$	δ_c(CH)
S_{20}	$\beta_{2,14,13}$	δ(OH)
S_{21}	$(2^{-1/2})(\beta_{3,13,2} - \beta_{1,13,2})$	δ(COH)
S_{22}	$(6^{-1/2})(\beta_{2,6,1} - \beta_{1,5,6} + \beta_{6,4,5} - \beta_{5,3,4} + \beta_{4,2,3} - \beta_{3,1,2})$	δ_a(CC)
S_{23}	$(12^{-1/2})(2\beta_{2,6,1} - \beta_{1,5,6} - \beta_{6,4,5} + 2\beta_{5,3,4} - \beta_{4,2,3} - \beta_{3,1,2})$	δ_b(CC)
S_{24}	$(4^{-1/2})(\beta_{1,5,6} - \beta_{6,4,5} + \beta_{4,2,3} - \beta_{3,1,2})$	δ_c(CC)
S_{25}	$(6^{-1/2})(2\beta_{1,12,10} - \beta_{12,11,10} - \beta_{1,11,10})$	δ(C=O)
S_{26}	$(2^{-1/2})(\beta_{1,11,10} - \beta_{12,11,10})$	δ_{al}(CH)
S_{27}	$(2^{-1/2})(\beta_{2,10,1} - \beta_{6,10,1})$	δ(CHO)
	A" symmetry	
S_{28}	$(6^{-1/2})(\tau_{1,6,5,4} - \tau_{6,5,4,3} + \tau_{5,4,3,2} - \tau_{4,3,2,1} + \tau_{3,2,1,6} - \tau_{2,1,6,5})$	τ_a(CC)
S_{29}	$(12^{-1/2})(2\tau_{1,6,5,4} - \tau_{6,5,4,3} - \tau_{5,4,3,2} + 2\tau_{4,3,2,1} - \tau_{3,2,1,6} - \tau_{2,1,6,5})$	τ_b(CC)
S_{30}	$(4^{-1/2})(\tau_{6,5,4,3} - \tau_{5,4,3,2} + \tau_{3,2,1,6} - \tau_{2,1,6,5})$	τ_c(CC)
S_{31}	$(2^{-1/2})(\tau_{14,13,3,2} + \tau_{14,13,3,1})$	τ(OH)
S_{32}	$(2^{-1/2})(\tau_{12,10,1,6} + \tau_{12,10,1,2})$	τ(C=O)
S_{33}	$(2^{-1/2})(\tau_{11,10,1,6} + \tau_{11,10,1,2})$	τ_{al}(CH)
S_{34}	$\gamma_{9,1,6,5}$	γ_a(CH)
S_{35}	$\gamma_{15,6,5,4}$	γ(C-Cl)
S_{36}	$(2^{-1/2})(\gamma_{8,5,4,3} + \tau_{7,2,3,4})$	γ_b(CH)
S_{37}	$(2^{-1/2})(\gamma_{8,5,4,3} - \tau_{7,2,3,4})$	γ_c(CH)
S_{38}	$\gamma_{13,1,2,3}$	γ(COH)
S_{39}	$\gamma_{10,6,1,2}$	γ(CHO)

[a] Abbreviations: ν = stretching, δ = in-plane bending, γ = out-of-plane bending, τ = torsion. See Table S2 for the atom numbering scheme; $r_{i,j}$ is the distance between atoms A_i and A_j; $\beta_{i,j,k}$ is the angle between vectors A_kA_i and A_kA_j; $\beta_{i,j,k,l}$ is the angle between vector A_kA_i and A_kA_j in the plane defined by A_i, A_k and A_l atoms; $\tau_{i,j,k,l}$ is the dihedral angle between the plane defined by A_i, A_j, A_k and the plane defined by A_j, A_k and A_l atoms; $\gamma_{i,j,k,l}$ is the angle between the vector A_kA_i and the plane defined by atoms A_j, A_k, A_l. The combinations [(+), (+)] and [(+), (−)] denote in-phase and in-opposite-phase couplings between coordinates of different types.

Table S2. Cartesian coordinates (Å) of the optimized structures of conformers **I-IV** of 5CSA.

Conformer **I**

C1	0.886542000	-0.502804000	0.000020000
C2	1.186884000	0.882082000	0.000000000
C3	0.135461000	1.807931000	0.000000000
C4	-1.179157000	1.371862000	0.000000000
C5	-1.472676000	0.001950000	0.000000000
C6	-0.454372000	-0.928439000	0.000000000
H7	0.371812000	2.864666000	0.000000000
H8	-1.988734000	2.091080000	0.000000000
H9	-0.677661000	-1.989198000	0.000000000
C10	1.958191000	-1.488795000	0.000000000
H11	1.644424000	-2.548575000	0.000000000
O12	3.151755000	-1.205889000	0.000000000
O13	2.446016000	1.338428000	0.000000000
H14	3.050326000	0.562209000	0.000000000
Cl15	-3.149857000	-0.523719000	0.000000000

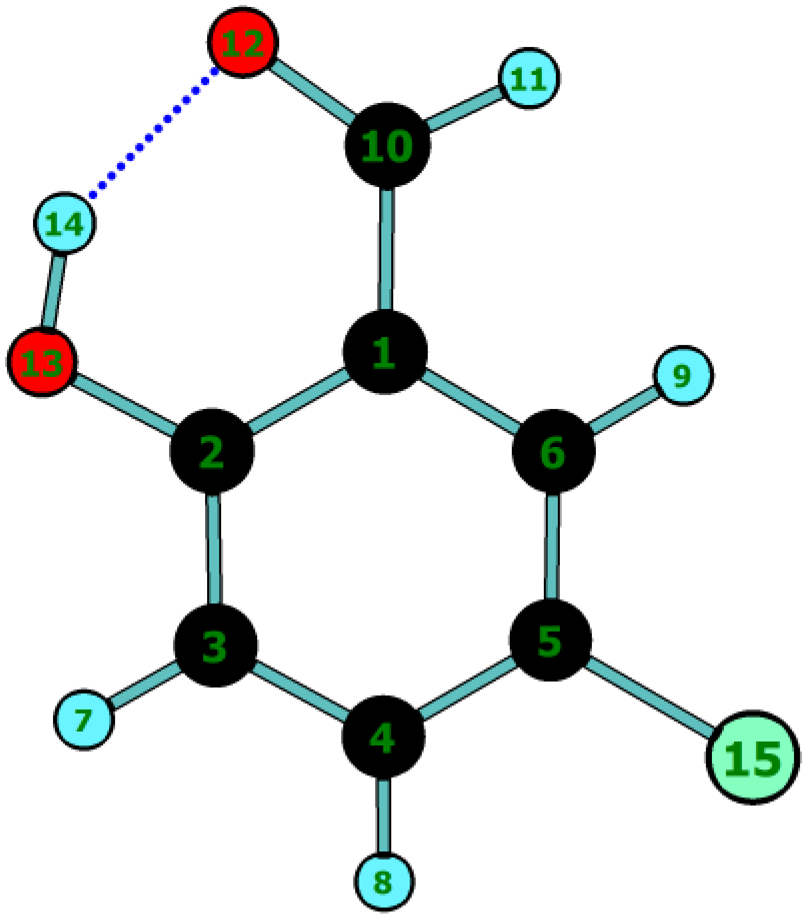

Conformer **II**

C1	-1.025860000	0.340403000	0.000000000
C2	-1.261005000	-1.045662000	0.000000000
C3	-0.183048000	-1.934071000	0.000000000
C4	1.121054000	-1.454896000	0.000000000
C5	1.352246000	-0.078943000	0.000000000
C6	0.293592000	0.811612000	0.000000000
H7	-0.362241000	-3.004883000	0.000000000
H8	1.954893000	-2.145423000	0.000000000
H9	0.455324000	1.882313000	0.000000000
C10	-2.144725000	1.314805000	0.000000000
H11	-3.157525000	0.877877000	0.000000000
O12	-1.984893000	2.517069000	0.000000000
O13	-2.554554000	-1.478244000	0.000000000
H14	-2.582651000	-2.440823000	0.000000000
Cl15	3.005545000	0.517697000	0.000008000

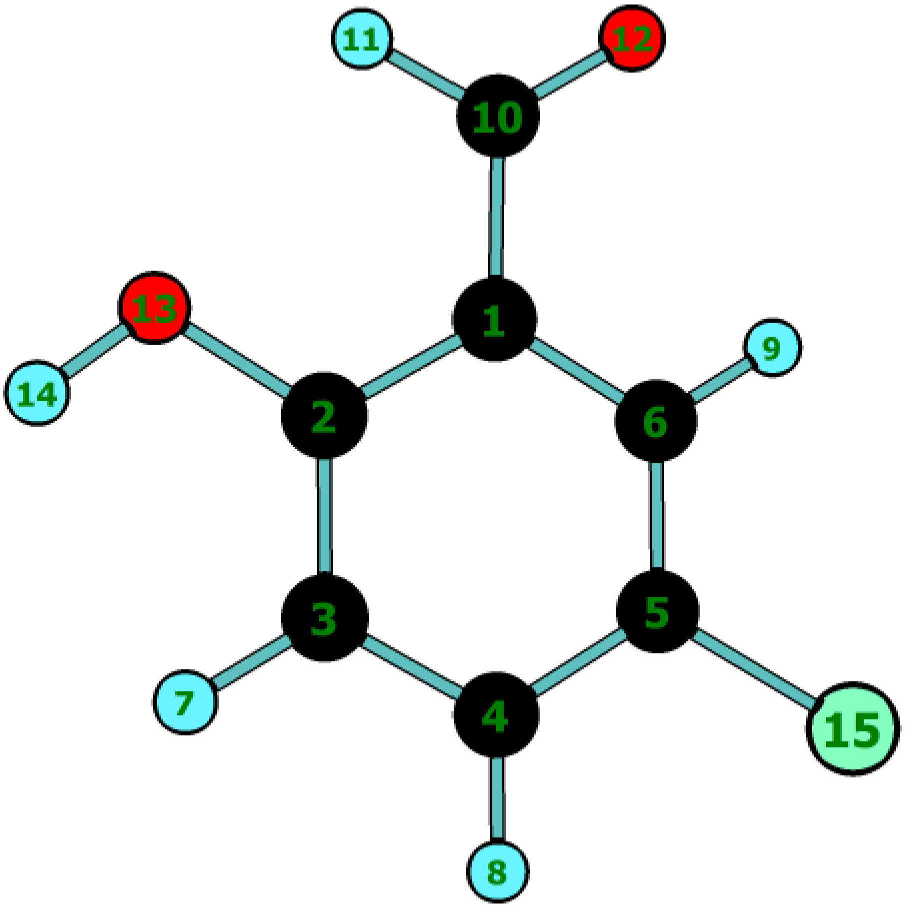

Conformer **III**

C1	1.030977000	0.328110000	0.000000000
C2	1.258668000	-1.062750000	0.000000000
C3	0.171111000	-1.942700000	0.000000000
C4	-1.124925000	-1.456174000	0.000000000
C5	-1.351576000	-0.074918000	0.000000000
C6	-0.292430000	0.807414000	0.000000000
H7	0.365802000	-3.007915000	0.000000000
H8	-1.963664000	-2.140823000	0.000000000
H9	-0.448327000	1.878634000	0.000000000
C10	2.129545000	1.310232000	0.000000000
H11	3.164043000	0.898225000	0.000000000
O12	1.985549000	2.512765000	0.000000000
O13	2.489668000	-1.644464000	0.000000000
H14	3.191440000	-0.985608000	0.000000000
Cl15	-3.002308000	0.526811000	0.000000000

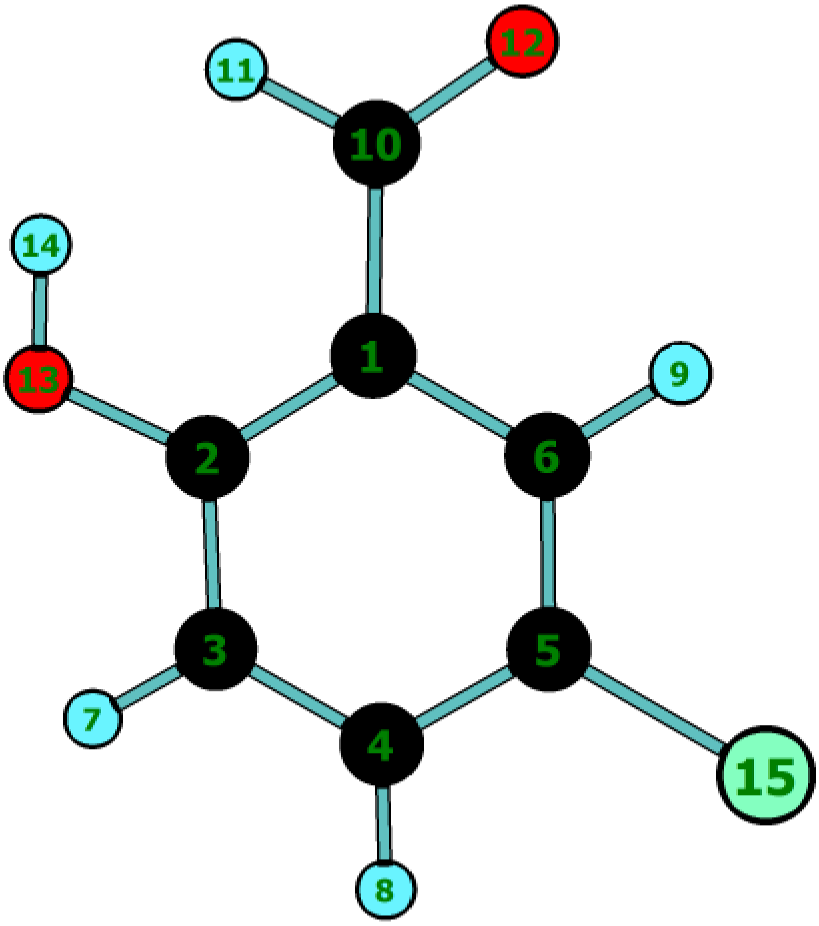

Conformer **IV**

C1	-0.912526000	-0.477362000	0.000000000
C2	-1.186869000	0.907566000	0.000000000
C3	-0.123275000	1.815804000	0.000000000
C4	1.195026000	1.376639000	0.000000000
C5	1.464510000	0.010452000	0.000000000
C6	0.423467000	-0.902458000	0.000000000
H7	-0.330795000	2.881649000	0.000000000
H8	2.007761000	2.091844000	0.000000000
H9	0.634864000	-1.965761000	0.000000000
C10	-1.948912000	-1.537829000	0.000000000
H11	-1.511040000	-2.560463000	0.000000000
O12	-3.146796000	-1.385989000	0.000000000
O13	-2.474342000	1.321442000	0.000000000
H14	-2.506580000	2.284807000	0.000000000
Cl15	3.129786000	-0.551328000	0.000000000

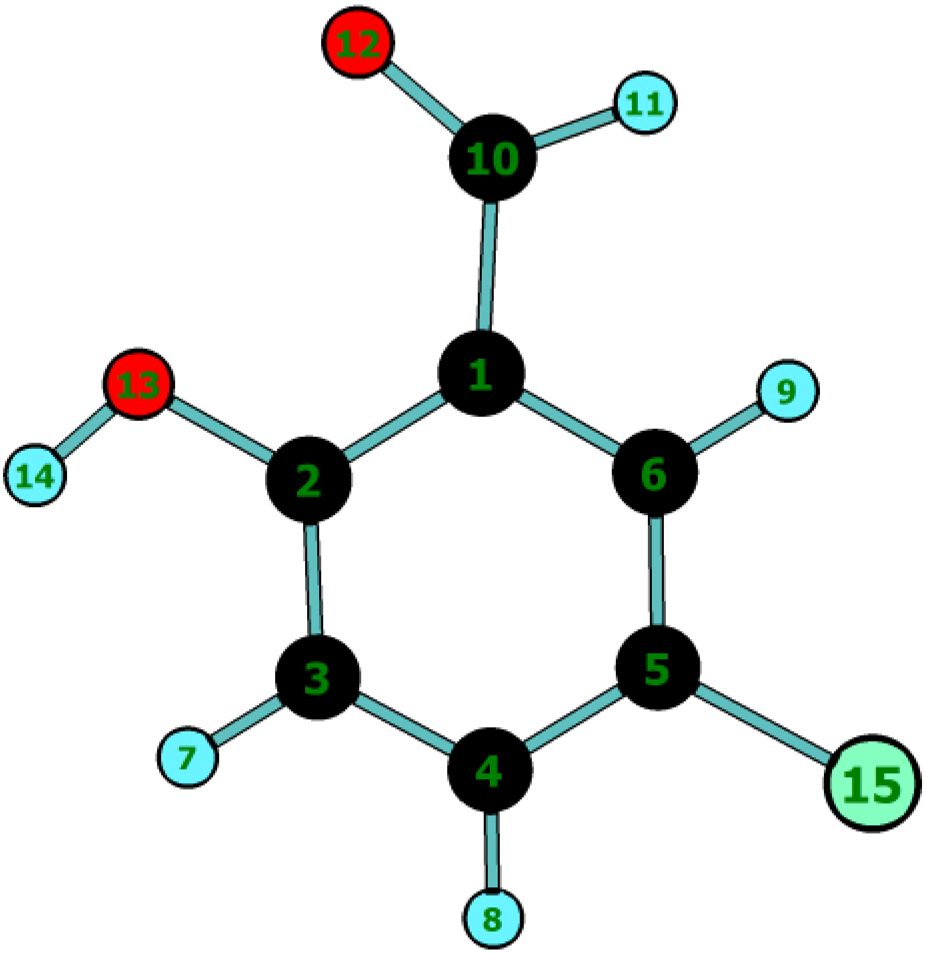

Supplement: Supplementary file 1 — jp2c03685_si_001.pdf [file jp2c03685_si_001.pdf]
